# Atg7 Regulates Brain Angiogenesis via NF-κB-Dependent IL-6 Production

**DOI:** 10.3390/ijms18050968

**Published:** 2017-05-03

**Authors:** Shi-Fang Zhuang, Dong-Xin Liu, Hui-Jie Wang, Shu-Hong Zhang, Jia-Yi Wei, Wen-Gang Fang, Ke Zhang, Liu Cao, Wei-Dong Zhao, Yu-Hua Chen

**Affiliations:** Department of Developmental Cell Biology, Key Laboratory of Cell Biology, Ministry of Public Health, and Key Laboratory of Medical Cell Biology, Ministry of Education, China Medical University, 77 Puhe Road, Shenbei New District, Shenyang 110122, China; zsfcmu@163.com (S.-F.Z.); dxliu@cmu.edu.cn (D.-X.L.); huihui825@139.com (H.-J.W.); zhangshuhong2002@163.com (S.-H.Z.); weijycmu@163.com (J.-Y.W.); fangwengang@126.com (W.-G.F.); kzhang@cmu.edu.cn (K.Z.); lcao@cmu.edu.cn (L.C.)

**Keywords:** Atg7, angiogenesis, brain microvascular endothelial cell, IL-6, NF-κB

## Abstract

The formation of brain vasculature is an essential step during central nervous system development. The molecular mechanism underlying brain angiogenesis remains incompletely understood. The role of Atg7, an autophagy-related protein, in brain angiogenesis was investigated in this study. We found that the microvessel density in mice brains with endothelial-specific knockout of Atg7 (Atg7 EKO) was significantly decreased compared to wild-type control. Consistently, in vitro angiogenesis assays showed that Atg7 knockdown impaired angiogenesis in brain microvascular endothelial cells. Further results indicated that knockdown of Atg7 reduced interleukin-6 (IL-6) expression in brain microvascular endothelial cells, which is mediated by NF-κB-dependent transcriptional control. Interestingly, exogenous IL-6 restored the impaired angiogenesis and reduced cell motility caused by Atg7 knockdown. These results demonstrated that Atg7 has proangiogenic activity in brain angiogenesis which is mediated by IL-6 production in a NF-κB-dependent manner.

## 1. Introduction

The brain relies on the steady supply of oxygen and nutrients from the blood stream of cerebral vessels. Neovascularization is present in the brain and occurs through the sprouting of new blood vessels from pre-existing vessels, called angiogenesis [1]. Angiogenesis in the brain is associated with a variety of neurological diseases, such as arteriovenous malformations, intracranial tumorigenesis, and neurodegenerative disorders [2]. In addition, brain angiogenesis is required for the functional recovery from ischemic stroke and traumatic brain injury [2]. Dissecting the molecular mechanism of brain angiogenesis will lead to a better understanding of the dynamic vascular changes in the brain under physiological and pathological conditions.

Angiogenesis is a tightly controlled process that is dependent on the balance between stimulating proangiogenic factors and inhibiting antiangiogenic factors. The well characterized proangiogenic factors include vascular endothelial growth factor (VEGF), fibroblast growth factors, placental growth factor, interleukins, etc., whereas thrombospondin, angiostatin, and endostatin are putative antiangiogenic factors [3]. The majority of identified angiogenic factors are produced by secretion or proteolytic cleavage. These factors act on neighboring endothelial cells to affect the angiogenesis via releasing into the extracellular environment [4]. The precise intracellular signaling responsible for the production of pro- and antiangiogenic factors remained to be investigated.

Atg7, an autophagy-related protein with homology to ubiquitin-activating enzyme E1, is essential for autophagy conjugation system and autophagosome formation [5,6]. Although emerging evidence links autophagy to angiogenesis [7], the role of Atg7 in angiogenesis in the brain remains unexplored. In this study, we used transgenic mice with endothelial-specific knockout of Atg7 (Atg7 EKO) [8] to explore the role of Atg7 in brain angiogenesis. We found Atg7 knockout significantly reduced microvessel density in the mice brain. Atg7 is positively associated with angiogenesis and migration of brain microvascular endothelial cells. Our further results demonstrated that the effect of Atg7 on brain angiogenesis is mediated by production of proangiogenic IL-6, which is dependent on nuclear factor NF-κB.

## 2. Results

### 2.1. Knockout of Atg7 Impaired Brain Angiogenesis in Mice

**Figure 1:**
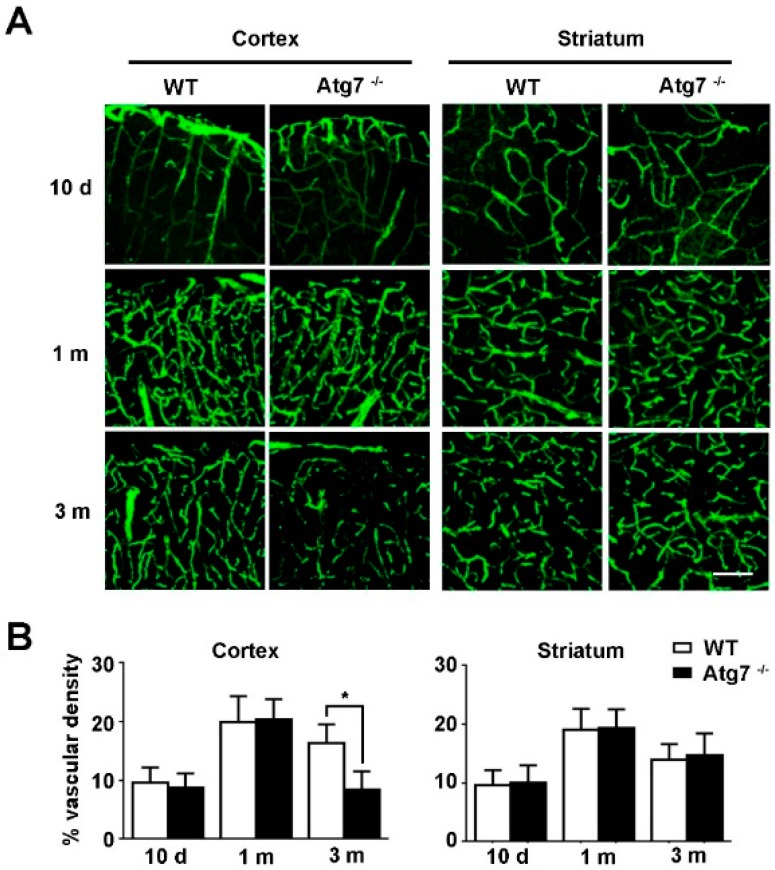
Brain microvessel changes in mice with endothelial-specific knockout of Atg7. (**A**) The tissue samples from brain cortex and striatum were obtained from transgenic mice with endothelial-specific knockout of Atg7 (Atg7^−/−^), with littermate wild-type mice as a control. Mice with different ages (10 days, 1 month, and 3 months) were analyzed, respectively. The brain slices were prepared using an oscillating microtome and immunofluorescence was performed with first antibody against platelet and endothelial cell adhesion molecule 1 (CD31, green). The stained slices were examined under confocal microscope (*n* = 10 brain slices from 3 different mice).The representative images from three independent experiments were presented. Scale, 100 μm; (**B**) The percentage of vascular density in (**A**) were calculated as follows: the vessel area is divided by the total image area and multiplied by 100. Images were analyzed by ImageJ software. * *p* < 0.05.

The mice harboring LoxP-flanked Atg7 were crossed with mice expressing Cre recombinase under the control of vascular endothelial cadherin (VE-cadherin) promoter, generating mice with endothelial-specific knockout of Atg7 (Atg7 EKO) [8]. The brain sections were obtained from the homozygous Atg7 EKO transgenic mice and the brain vessels were analyzed by immunofluorescence. The results showed that the microvessel density at brain cortex in three-month-old Atg7 EKO mice was significantly reduced compared to the littermate wild-type mice, without detectable changes at the age of 10 days and 1 month (Figure 1, left panel). In contrast, the microvessel density at striatum were not affected by Atg7 depletion (Figure 1, right panel). These results revealed the inhibitory effect of Atg7 knockout on brain angiogenesis, which is developmental stage-specific and brain site-specific.

### 2.2. Atg7 Knockdown Reduced Angiogenesis of Brain Endothelial Cells

**Figure 2:**
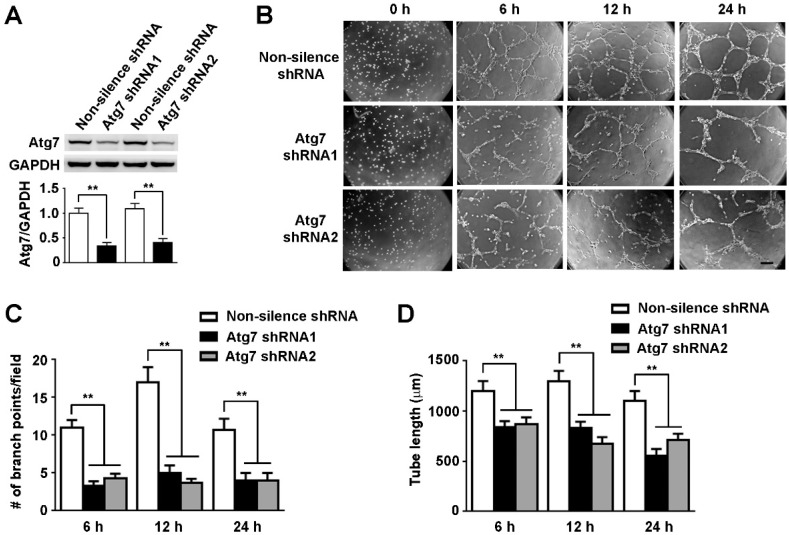
Knockdown of Atg7 inhibited angiogenesis of brain microvascular endothelial cells. (**A**) Human brain microvascular endothelial cells (HBMEC) were stably transfected with Atg7-specific shRNA construct, Atg7 shRNA1, and Atg7 shRNA2, respectively. HBMEC stably transfected with non-silencing shRNA were served as the control. Then the protein levels of Atg7 were examined by western blot, with glyceraldehyde-3-phosphate dehydrogenase (GAPDH) as the loading control. The relative expression level of Atg7 and Atg7/GAPDH were calculated by measuring the band intensity using ImageJ software. ** *p* < 0.01; (**B**) Tube formation assays were performed with HBMEC stably transfected with Atg7 shRNA1 and Atg7 shRNA2, respectively, with non-silencing shRNA as the control. Then the images were captured under an inverted microscope at indicated times. The representative images from three independent experiments were shown. Scale, 200 μm; (**C**,**D**) To quantify the results of tube formation assays in (**B**), the number of branch points were counted and the tube length were calculated. ** *p* < 0.01.

Next, cultured human brain microvascular endothelial cells (HBMEC), were used to test the effect of Atg7 on angiogenesis. Stable HBMEC cell lines transfected with Atg7-specific shRNA were constructed, with empty vector as a control. The knockdown effect was analyzed by western blot and the results showed that the levels of Atg7 were significantly decreased compared to non-silencing shRNA control (Figure 2A). Then the stable HBMEC cell lines with silenced Atg7 were subjected to in vitro tube formation assay to test their angiogenesis ability. We found that knockdown of Atg7 effectively attenuated the in vitro angiogenesis of HBMEC compared to non-silencing control (Figure 2B). The branch points and tube length were significantly reduced upon Atg7 knockdown (Figure 2C,D). These results indicated that depletion of Atg7 inhibited angiogenesis of brain endothelial cells, which is in line with the results from Atg7 EKO mice (Figure 1).

### 2.3. IL-6 Reduction Accounts for the Impaired Angiogenesis Induced by Atg7 Depletion

**Figure 3:**
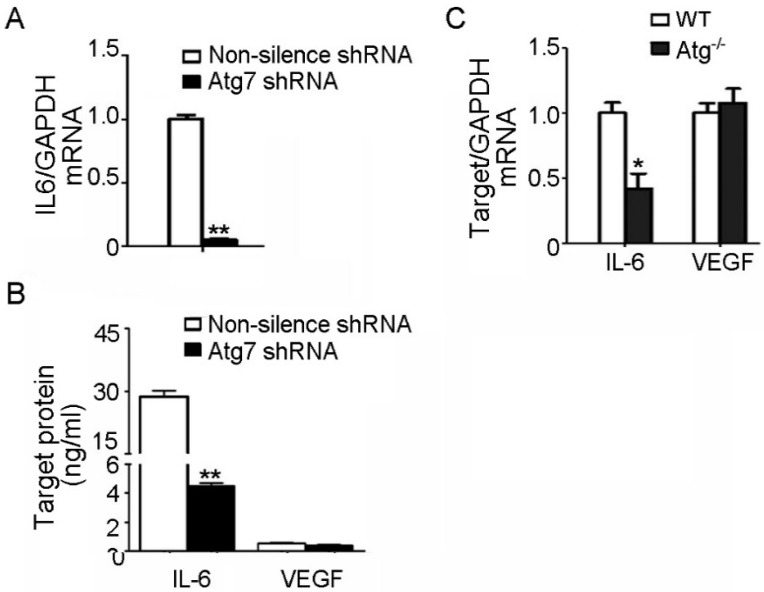
Atg7 knockdown reduced IL-6 production in brain endothelial cells. (**A**) The mRNA levels of IL-6 in the HBMEC transfected with Atg7 shRNA1 were determined by real time RT-PCR. HBMEC transfected with non-silencing shRNA were used as control. ** *p <* 0.01; (**B**) The concentration of IL-6 and vascular endothelial growth factor (VEGF) in the supernatant of HBMEC transfected with Atg7 shRNA1 were determined by ELISA. ** *p <* 0.01; (**C**) The mRNA levels of IL-6 and VEGF in the brain cortex from the three-month-old Atg7 endothelial-specific knockout mice were determined by real time RT-PCR, with littermate wild-type mice as control. * *p* < 0.05.

Our further results showed that the expression of IL-6, a prominent proangiogenic factor involved in angiogenesis during tumor progression [9], was significantly decreased in Atg7-silenced HBMEC compared to the control (Figure 3A). The paracrine effects of IL-6 are achieved by secretion [9], thus the secreted IL-6 in the supernatant of Atg7-silenced HBMEC were determined by ELISA assay. The results showed that depletion of Atg7 in HBMEC led to a significant reduction in IL-6 secretion compared to the non-silencing control (Figure 3B). In contrast, VEGF, a well-known factor with pro-angiogenic activity [10], remained unchanged with Atg7 knockdown (Figure 3B). Then, the expression of IL-6 and VEGF were examined by real-time RT-PCR in the brain cortex. The results showed that IL-6 expression was significantly decreased in Atg7 EKO mice compared to wild-type mice, whereas VEGF remained unchanged (Figure 3C).

**Figure 4:**
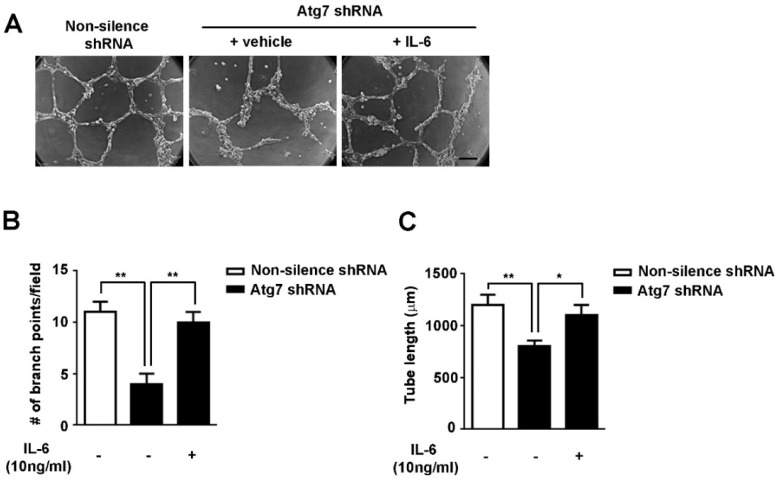
Exogenous addition of IL-6 restored the impaired angiogenesis in brain endothelial cells with Atg7 knockdown. (**A**) Tube formation assays were performed with the indicated HBMEC in the absence (vehicle) or presence of IL-6 (10 ng/mL). The representative images from three independent experiments were presented. Scale, 200 μm; (**B**,**C**) To quantify the results in (**A**), the number of branch points (**B**) and the tube lengths were calculated (**C**). * *p* < 0.05, ** *p* < 0.01.

To test whether IL-6 is associated with the impaired angiogenesis caused by Atg7 depletion, tube formation assays were performed with Atg7-silenced HBMEC in the presence of recombinant IL-6. The results showed that the exogenous applied IL-6 (10 ng/mL) efficiently restored the inhibition of angiogenesis by Atg7 knockdown (Figure 4A). The reduced branch points and tube length induced by Atg7 knockdown were significantly restored by recombinant IL-6 (Figure 4B,C). These results suggested that the impaired angiogenesis induced by Atg7 depletion is mediated by reduced IL-6 production in brain endothelial cells. In other words, depletion of Atg7 reduced IL-6 expression to attenuate the angiogenesis of brain endothelial cells.

### 2.4. Atg7 Modulates Migration of Brain Endothelial Cells via IL-6

**Figure 5:**
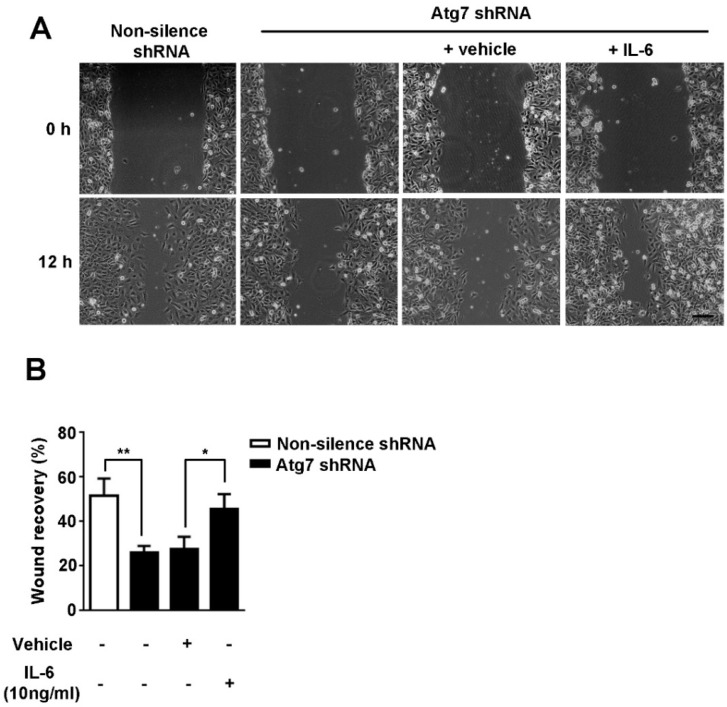
Knockdown of Atg7 inhibited migration of brain microvascular endothelial cell, which was restored by exogenous IL-6. (**A**) The scratch wound assays were performed using the indicated transfected HBMEC in the absence (vehicle) or presence of IL-6 (10 ng/mL). The representative images at indicated times from three independent experiments were shown. Scale, 200 μm; (**B**) To quantify the results in (**A**), the wound recover rates were calculated by ImageJ software. * *p* < 0.05, ** *p* < 0.01.

It is known that proliferation and migration of endothelial cells is associated with the angiogenesis process. Knockdown of Atg7 did not affect proliferation of HBMEC [11] prompts us to measure the cell migration ability in Atg7-depleted HBMEC by scratch wound assay. We found the cell migration of Atg7-silenced HBMEC was significantly inhibited compared to the non-silencing control (Figure 5A, left two columns; Figure 5B). The result suggested that Atg7 is associated with the cell migration, but not proliferation, of brain endothelial cells to regulate its angiogenesis. Our further results showed that addition of exogenous IL-6 was able to restore the decreased migration in Atg7-silenced HBMEC (Figure 5A, right two columns; Figure 5B). These results indicated that Atg7 regulates the migration of brain endothelial cells, dependent on IL-6 production.

### 2.5. Atg7 Regulates IL-6 Transcription through Nuclear Factor NF-κB

**Figure 6:**
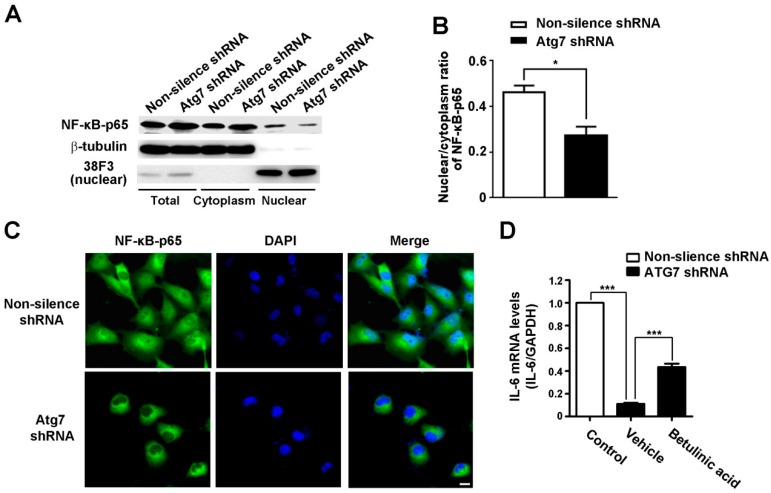
Interleukin-6 promoted cell migration to rescue angiogenesis in the Atg7-knockdown brain microvascular endothelial cells. (**A**) The nuclear and cytoplasmic extracts were obtained in HBMEC stably transfected with Atg7 shRNA1 (Atg7 KD). Then the expression of the NF-κB p65 subunit was analyzed by western blot. β-tubulin and 38F3 were detected as marker proteins for cytoplasm and nuclear, respectively. HBMEC transfected with non-silencing shRNA were used as control. The representative images were from three independent experiments; (**B**) To quantify the results in (**A**), the band intensities of p65 in nuclear and cytoplasm fractions were measured by ImageJ software and the nuclear to cytoplasm ratios of p65 was calculated. * *p* < 0.05; (**C**) HBMEC stably transfected with Atg7 shRNA1 were seeded on coverslips and immunofluorescence was conducted with antibody against p65 (green). DAPI (blue) was used for counterstaining. HBMEC transfected with non-silencing shRNA were served as a control. Scale, 20 μm; (**D**) The mRNA levels of IL-6 in the HBMEC transfected with Atg7 shRNA1 were determined by real time RT-PCR, with HBMEC transfected with non-silencing shRNA as a control. When indicated, the cells were incubated with NF-κB agonist and betulinic acid (10 μg/mL) for 2 h. *** *p* < 0.001.

Our real-time RT-PCR results showed that the mRNA level of IL-6 was significantly decreased in Atg7-silenced HBMEC (Figure 3A,B), suggesting IL-6 was transcriptionally controlled by Atg7. It has been shown that transcription factor NF-κB is a key regulator of IL-6 transcription [12]. Thus, we analyzed the nuclear localization of p65, a subunit of NF-κB transcription complex, to evaluate the activation of NF-κB in Atg7-silenced HBMEC. The nuclear and cytoplasmic fractions were extracted and the expression of p65 was measured by western blot (Figure 6A). The nuclear to cytoplasm ratios of p65 was calculated and the results showed the nuclear distribution of p65 was significantly reduced by Atg7 knockdown (Figure 6B). Consistently, immunofluorescence results showed that Atg7 knockdown significantly reduced the nuclear localization of p65 (Figure 6C). Further results showed that NF-κB agonist, betulinic acid, effectively restored the reduction of IL-6 caused by Atg7 knockdown (Figure 6D). These results demonstrated that Atg7 is able to regulate IL-6 transcription via nuclear translocation of NF-κB in brain endothelial cells.

## 3. Discussion

Autophagy is a catabolic recycling pathway conserved from yeast to mammals. Increasing evidence indicated that autophagy is involved in vertebrate development by modulating various cellular process including programmed cell death, proliferation, survival, differentiation, etc. [13,14]. Here, we found that Atg7, an autophagy-related protein, is associated with the angiogenesis in the brain. Endothelial-specific knockout of Atg7 significantly reduced microvessel intensity in the brains of three-month-old mice. The RNA interference-mediated knockdown of Atg7 in brain endothelial cells reduced its in vitro angiogenesis. These results, for the first time, revealed the proangiogenic role of Atg7 in brain angiogenesis, indicating that autophagy is associated with brain angiogenesis.

In line with our findings, a recent study showed that knockdown of Atg7 in umbilical vein endothelial cells attenuated in vitro capillary tube formation [15], suggesting that Atg7 is not only associated with angiogenesis in the brain, but also involved in angiogenesis of peripheral vessels. Regarding the underlying mechanism of Atg7-regulated angiogenesis, we found that Atg7 depletion led to decreased IL-6 secretion in brain endothelial cells. IL-6 is an important inflammatory mediator and the effects of IL-6 on angiogenesis during tumor progression have been demonstrated [9]. In contrast, the secretion of VEGF, a well-known proangiogenic factor [10], was not affected by Atg7. Moreover, exogenous application of recombinant IL-6 could restore the attenuated angiogenesis induced by Atg7 knockdown in brain endothelial cells. Thus our results pointed out that Atg7 facilitates angiogenesis in the brain through secretion of proangiogenic IL-6, but not VEGF.

With whole-genome transcriptional profiling, Stephenson et al. identified a set of differentially expressed genes in Atg5-deficient thymocytes [16], which demonstrated that autophagy is associated with transcriptional controls. Here, we found IL-6 mRNA levels were reduced upon Atg7 knockdown in brain endothelial cells. Previous studies identified NF-κB as the central transcription factor mediating IL-6 expression during vascular inflammation [12]. Our further results demonstrated that Atg7 is able to regulate transcription of IL-6 via nuclear factor NF-κB. These findings thus unveil the novel role of Atg7 in transcriptional regulation beyond its well-known function in autophagy.

In a previous study with endothelial knockout of Atg7, Torisu et al. found that Atg7 knockout has little effect on the retinal vasculature in eight-day-old mice [17]. We think this discrepancy was caused by the age of mice because we found decreased brain vascular density in Atg7 EKO mice was prominent at 3 months of age (Figure 1A,B) whereas it did not show any changes at 10 days of age. A recent study with in vivo imaging demonstrated that the brain microvessel formation continued until one month after birth in mice [18]. Our results showed that Atg7 knockout has little effect on brain vascular density in 10-day-old and 1-month-old mice, and we consider the possible reason is that Atg7 was not an indispensable regulatory factor for brain microvessel formation in the first month after birth. In contrast, we found Atg7 knockout reduced the brain vascular density in three-month-old mice. Given that Atg7 is associated with in vitro angiogenesis of brain endothelial cells, we concluded that Atg7 could facilitate adult angiogenesis in the brain for the maintenance of brain vasculature in adult mice.

## 4. Materials and Methods

### 4.1. Animal Experiments

The construction of mice with Atg7 conditional knockout in endothelial cells (Atg7 EKO) and the genotyping procedure were described previously [5,19,20]. All the animal study experiments were approved by Animal Experimentation Ethics Committee of China Medical University (14031, 29 April 2017).

### 4.2. Cell Culture

Human brain microvascular endothelial cell line (HBMEC) was a kind gift from Dr. Kwang Sik Kim (Johns Hopkins University School of Medicine, Baltimore, MD, USA). HBMEC were cultured in RPMI 1640 medium, supplemented with 10% fetal bovine serum (HyClone, Logan, UT, USA), 10% Nu-serum (BD Biosciences, San Jose, CA, USA), 2 mM glutamine, 1 mM sodium pyruvate, 1× nonessential amino acid, and 1× minimal essential medium (MEM) vitamin. The cells were incubated at 37 °C in a 5% CO_2_, 95% air humidified atmosphere.

### 4.3. RNA Interference

The small hairpin RNA (shRNA) sequence targeting human Atg7 (NM_006395) corresponding to the coding region, GGTCAAAGGACGAAGATAA (781–799), was built by GenScript’s shRNA design center (GenScript, Piscataway, NJ, USA). A non-silencing shRNA sequence (TTCTCCGAACGTGTCACGT) (24898658) was used as a control. For establishment of stable cell line with knockdown of Atg7, the shRNA sequences were inserted into pRNA-U6.1/Neo vector (GenScript). Recombinant shRNA plasmids were transfected into HBMEC using Lipofectamine2000 (Invitrogen, Carlsbad, CA, USA) and stable transfectants were selected by G418 (300 μg/mL; Invitrogen).

### 4.4. Transcription (RT)-PCR

The mice were sacrificed and the brain cortexes were collected. Then TRI Reagent (1 mL per 100 mg of tissue, Sigma-Aldrich, St. Louis, MO, USA) were added and the tissues were homogenized. The total RNA of brain tissues were extracted according to manufacturer’s instructions. The total RNA of cells were extracted with TRI Reagent and reverse transcribed with Moloney murine leukemia virus (M-MLV) reverse transcriptase (Promega, Madison, WI, USA). Real-time PCR was performed on an ABI 7500 real-time PCR system (Thermo Fisher Scientific, Waltham, MA, USA) with a SYBR premix Ex Taq kit (Takara Biotechnology, Dalian, China), according to the manufacturer’s instructions. The primer sequences for IL-6 were ACACAGACAGCCACTCACCTC (forward) and AGCATCCATCTTTTTCAGCCA (reverse); Primers for GAPDH were GAAGGTGAAGGTCGGAGTC (forward) and GAAGATGGTGATGGGATTTC (reverse). Real-time PCR products were analyzed on agarose gel electrophoresis and verified by DNA sequencing. The comparative cycle threshold (CT) method was used to calculate the relative expression level of IL-6, with GAPDH as the internal control.

### 4.5. Immunofluorescence

For immunostaining of brain slices, the mice were anesthetized with sodium pentobarbital anesthesia and perfused transcardially with 1× PBS, followed by 4% paraformaldehyde for 1 h. Mice brains were post-fixed overnight and then coronal sections (100 µm) were obtained and incubated in 5% donkey serum in PBS for 1 h. The anti-CD31 antibody (Abcam, Cambridge, UK) was diluted in PBS containing 1% donkey serum and incubated with brain sections overnight at 4 °C. The sections were subjected to anti-rabbit-Cy3 secondary antibody (1:250, Jackson, West Grove, PA, USA). Then the brain sections were mounted and visualized under confocal laser scanning microscope (Zeiss 880, Oberkochen, Germany). For immunostaining of NF-κB in HBMEC, the cells were fixed and incubated with antibody against p65 of NF-κB (Abcam).

### 4.6. Western Blot

Cells were washed twice with ice-cold PBS and prepared with radioimmunoprecipitation assay (RIPA) buffer (50 mM Tris-HCl, 150 mM NaCl, 1% NP-40, 0.5% deoxycholate, 0.1% sodium dodecylsulfate) containing protease inhibitor cocktail (Roche, Indianapolis, IN, USA). The samples were loaded and separated by SDS-PAGE, and then transferred to the PVDF membrane (Millipore, Billerica, MA, USA). The PVDF membrane was then blocked with 5% non-fat milk and probed with the first antibody against Atg7, GAPDH, p65, β-tubulin, or 38F3 (Abcam) at 4 °C overnight. The blots were then incubated with an Horse Reddish Peroxidase (HRP)-conjugated secondary antibody (Santa Cruz, Dallas, TX, USA) for 1 h at room temperature. Immunoreactive bands were visualized by the SuperSignal West Pico Chemiluminescent Substrate (Pierce Chemical, Rockford, IL, USA).

### 4.7. Enzyme-Linked Immunosorbent Assay (ELISA)

We inoculated the same number of cells in the 35-mm dish and cultured them until 80% confluence with normal culture media. Then the cells were replaced with serum-free media and incubated for 24 h. The supernatant of cells was collected and the concentrations of IL-6 and VEGF were determined with ELISA kits (IBL, Gunma, Japan) according to the manufacturer’s instructions.

### 4.8. Tube Formation Assay

Tube formation was performed as described previously [21]. Briefly, 1 × 10^5^ HBMEC were seeded into the wells of a 24-well plate coated with growth factor-reduced Matrigel (BD Biosciences). Images were acquired on an inverted microscope (Axiovert 40, Carl Zeiss, Oberkochen, Germany) after plating at indicated time points. Three independent experiments were performed with each in duplicates. The branch points and tube length were calculated with ImageJ software (version 1.50, Bethesda, MD, USA).

### 4.9. Scratch Wound Assay

Scratch wound assay was conducted as described previously [21]. Briefly, HBMEC cultured in 35-mm dishes were starved for 12 h in RPMI 1640 with 1% FBS. After starvation, a scratch wound was made by creating a linear cell-free region using a pipette tip. Detached cells were removed by washing cells twice with PBS and a smooth edge of the scratch was obtained. Then the cells were return back to incubator. The progress of cell migration into the scratch was photographed at 0, 4, 8, and 12 h by an inverted microscope (Axiovert 40, Carl Zeiss, Oberkochen, Germany). Results are shown as percent wound recovery which was calculated as follows: (Wound Area (initial)–Wound Area (final))/Wound area (initial) × 100. Data are acquired from three independent experiments with each in duplicate.

### 4.10. Statistical Analysis

All quantitative data are expressed as means ± S.D. of three independent experiments. The statistical analysis was performed using one-way ANOVA to analyze data for more than two groups. Student’s *t*-test was used for data with two groups.

## 5. Conclusions

In summary, we found that Atg7 is positively associated with the brain microvasculature development. Atg7 regulates IL-6 production via NF-κB to modulate brain angiogenesis. These findings established Atg7 as a novel regulatory molecule contributing to angiogenesis in the brain.
